# Don’t Shoot the Messenger? A Morality- and Gender-Based Model of Reactions to Negative Workplace Gossip

**DOI:** 10.1007/s10551-023-05355-7

**Published:** 2023-03-04

**Authors:** Maria Kakarika, Shiva Taghavi, Helena V. González-Gómez

**Affiliations:** 1 Durham University Business School, Durham, UK; 2 NEOMA Business School, Mont-Saint-Aignan, France

**Keywords:** Gossip, Morality, Sanctioning, Gender, Attribution

## Abstract

We conducted three studies to examine how the recipients of negative workplace gossip judge the gossip sender’s morality and how they respond behaviorally. Study 1 provided experimental evidence that gossip recipients perceive senders as low in morality, with female recipients rating the sender’s morality more negatively than male recipients. In a follow-up experiment (Study 2), we further found that perceived low morality translates into behavioral responses in the form of career-related sanctions by the recipient on the gossip sender. A critical incident study (Study 3) enhanced the external validity and extended the moderated mediation model by showing that gossip recipients also penalize senders with social exclusion. We discuss the implications for practice and research on negative workplace gossip, gender differences in attributions of morality, and gossip recipients’ behavioral responses.

**Supplementary Information:**

The online version contains supplementary material available at 10.1007/s10551-023-05355-7.

My dear Arthur, I never talk scandal. I only talk gossip. What is the difference between scandal and gossip? Oh! Gossip is charming! History is merely gossip. But scandal is gossip made tedious by morality.

– Oscar Wilde, Lady Windermere's Fan

Research has found that gossip is a significant part of people’s spoken general communication at work (Dunbar, 2004) and a natural part of socializing in organizations (Kniffin & Wilson, 2010). Nevertheless, research on this complex social phenomenon has been insufficient (Brady et al., 2017; Wu et al., 2018) and inconclusive (Dores Cruz et al., 2021). Within this small but growing body of literature, workplace gossip has been defined as the informal and evaluative talk among organizational members about another member who is not present (Kurland & Pelled, 2000). Even though positive gossip and negative gossip may be equally likely to occur (Dores Cruz et al., 2021), a solid body of previous research indicates that people are more sensitive to and more interested in hearing and sharing negative rather than positive gossip (e.g., Bosson et al, 2006; Robbins & Karan, 2020). We therefore focus on negative gossip in this paper.

In effect, a gossip episode involves three important parties: the *gossip sender,* who communicates information about the *target* to the *gossip recipient*, potentially influencing the target’s social reputation*.* Although gossip can have important social consequences for all three parties involved (Dores Cruz et al., 2021; Michelson et al., 2010), existing literature has focused mostly on the gossip target (e.g., Cheng et al., 2020; Feinberg et al., 2014; Sommerfeld et al., 2008; Wu et al., 2018; Zinko & Rubin, 2015) and to a lesser extent on the sender (Brady et al., 2017; Ellwardt et al., 2012a, 2012b, 2012c; Ellwardt et al., 2012a, 2012b, 2012c; Grosser et al., 2010). The recipient’s perspective, however, has received less attention (Bai et al., 2020; Kuo et al., 2015; Martinescu et al., 2014). This gap is problematic because gossip does not solely influence how the target is perceived (Feinberg et al., 2014; Zinko & Rubin, 2015); it also has social consequences for the gossip sender. Examining how gossip is interpreted by the recipient can elucidate these consequences (Lee & Barnes, 2021).

Yet, few studies to date have theoretically considered or empirically investigated these consequences from the recipient’s perspective (Lee & Barnes, 2021; see Sun et al., 2022 for a review). Recipients’ responses can take the form of dispositional judgments of the gossip sender and behavioral reactions. Research supports the notion that negative gossip about a third party falls in the moral domain (Peters & Kashima, 2014, p. 9) and is morally condemned and frowned upon (Ben-Ze'ev & Goodman, 1994; Emler, 1994; Foster, 2004). Unless the sender’s motive is perceived as prosocial, that is, as an attempt to protect the group from dominant norm violators (Wilson et al., 2000) and share diagnostic information about the target’s own morality (Peters & Kashima, 2015), gossip may be considered a form of norm violation and deviant behavior (Robinson & Bennett, 1995) because of the societal moral codes that discourage it (Ben-Ze'ev & Goodman, 1994; Levin & Arluke, 1987). Due to such a generalized notion that gossip is immoral behavior, gossip recipients are likely to ‘blame’ senders for the negative gossip they spread and consequently sanction them. For example, recipients may socially disapprove (Beersma & Van Kleef, 2012), dislike and distrust the gossip sender (Ellwardt et al., 2012; Farley, 2011; Turner et al., 2003), and engage in social undermining (Duffy et al., 2002) and social exclusion behaviors (Ellwardt et al., 2012a, 2012b, 2012c). Despite the importance of this logic, our understanding of how negative workplace gossip influences recipients’ moral attributions and behavior toward the sender remains limited and lacks a clear theoretical framework (Lee & Barnes, 2021).

Another question that has remained unanswered is whether gossip recipients’ gender plays a role in their responses to the sender. Extant research has argued that there are gender differences in the way individuals experience gossip (Leaper & Holliday, 1995; Michelson & Mouly, 2000; Watson, 2012) and that women engage in more gossip behavior than men (Robbins & Karan, 2020); however, whether gossip recipients’ judgments of the sender’s morality differ by gender has been understudied. Research on gender roles and socialization (Eagly, 1987; Eagly & Steffen, 1984; Eagly & Wood, 1991) indicates that women are socially expected to be more communal and men more agentic. One would thus expect women to exemplify the warmth dimension when evaluating the gossip senders and rate their morality less severely than men. By contrast, research on gender differences in harm aversion and deontological inclinations (Armstrong et al., 2019; Friesdorf et al., 2015) suggests that women would morally condemn the gossip sender more than men. Clearly, the theoretical directions remain inconsistent when it comes to explaining how male and female gossip recipients rate and behave toward senders.

To address these gaps, we propose and test a theoretical model of judgments of the gossip sender’s morality as the proximal consequence of negative gossip (Fig. 1). In turn, negative judgments trigger sanctioning responses to the gossip sender in the form of career-related penalties and social exclusion. We further test how the effects of negative workplace gossip are shaped by the recipient’s gender.

**Fig. 1:**
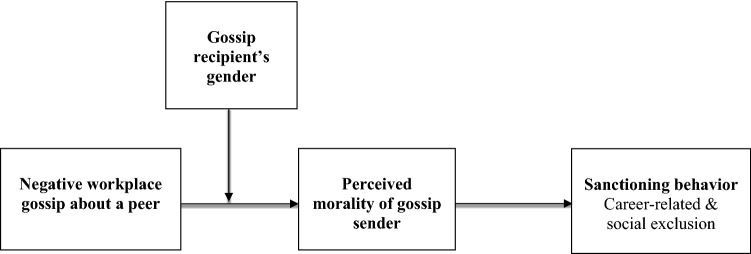
Overall hypothesized model

Our theoretical model contributes to the literature on workplace gossip in several ways. First, we develop the gossip recipient’s perspective of the gossip literature (Hauke & Abele, 2020) through a morality lens (Peters & Kashima, 2014, 2015), responding to calls to scrutinize the relationships between gossip senders and recipients (Bai et al., 2020). Based on attribution theory (Heider, 1958; Kelley & Michela, 1980), we integrate gossip research with the moral psychology literature (Funder, 2004) and treat negative gossip as a workplace event that elicits subsequent attributions of morality. Our study contextualizes and expands on findings by Peters and Kashima (2014), who examined the *correlation* between recipients’ perceived usefulness of gossip (both positive and negative) and such attributions of morality within a student context. We focus instead on typical negative gossip and its *causal* effects on attributions of senders’ morality within a work context, regardless of the gossip’s perceived utility. We thus add to our understanding of how negative gossip is interpreted and how senders are perceived by the recipient, advancing the workplace gossip literature and shedding light on gossip recipients’ responses.

Second, our study is unique in introducing the gossip recipient’s gender as an important moderator of the relationship between gossip and dispositional attributions. Our work integrates insights from gender role stereotypes and role congruity theory (e.g., Eagly, 1987) with research on gender differences in moral reasoning (Friesdorf et al., 2015) to understand the differences between men and women in terms of their judgments of the gossip sender’s morality. As such, we move beyond comparisons between women and men in terms of the frequency (Robbins & Karan, 2020) and subject of their gossip (Eckhaus & Ben-Hador, 2019), or gossip about same-sex others (McAndrew et al., 2007). Rather, we build on this ongoing conversation about workplace gossip and the sender’s gender (Eckhaus & Ben-Hador, 2019), and add important insights into how individual responses to receiving negative workplace gossip vary based on the gender of the recipient.

Third, our research advances our understanding of the social consequences for the gossip sender by examining the recipients’ behavioral responses. We uncover both social and career-related sanctions as important behavioral reactions on the part of the gossip recipients. Previous studies have examined recipients’ sanctioning of those who do not spread gossip on a norm-violating target (e.g., Wilson et al., 2000). We go further by providing a nuanced view of how gossip senders may be penalized at work by their peers (c.f. Grosser et al., 2010), shedding light on the unclear relationship between gossip engagement and career-related consequences (Sun et al., 2022). We identify negative judgments of the sender’s morality as an important mechanism that explains these career-related and social consequences for the gossip sender, regardless of the sender’s motives (c.f., Beersma & Van Kleef, 2012; Hartung et al., 2019; Wilson et al., 2000). We thus respond to the call of Brady et al. (2017) for research that delineates the processes underlying gossip and employee work outcomes.

Overall, our study increases the understanding of workplace gossip by taking a novel morality- and gender-based approach to how negative gossip can backfire on gossip senders. Our methodology combined two experimental studies with a critical incident field study to ensure both causal claims and external validity.

## Theoretical Background and Hypotheses

### Judgments of the Gossip Sender’s Morality

According to attribution theory, individuals continuously attribute causes to the actions and behaviors of others to make sense of their reality (Heider, 1958; Kelley & Michela, 1980). We suggest that during this process they are likely to evaluate the spreading of negative information about a target that is not present as a morally wrong behavior that violates norms (Robinson & Bennett, 1995). This expectation is consistent with generalized societal notions of gossip as something bad and immoral (Ben-Ze'ev & Goodman, 1994). Research on negative gossip converges on the idea that the sender’s motive is often not benevolent (see Kurland & Pelled, 2000). Although gossip behavior at times conveys useful information (Peters & Kashima, 2014, 2015) that condemns a norm violation, thereby serving the group (Beersma & Van Kleef, 2012; Dores Cruz et al., 2019; Wilson et al., 2000), it has great potential to violate the target’s privacy (Bies, 2001; Cheng et al., 2020) and damage their reputation (Emler, 1994; Tebbutt, 1995; Wert & Salovey, 2004). Wilson et al. (2000) found that the sender’s reputation was especially damaged when their motive was self-serving, whereas the evaluation of the sender was neutral when referring to a rule-breaking event. In addition, research shows that engaging in negative gossip may indicate that the sender is jealous or envious (Wert & Salovey, 2004) and acts deliberately to gain attention and power (Kurland & Pelled, 2000). Gossip recipients may also suspect that they could become future targets of the sender’s gossip (Ellwardt et al., 2012a, 2012b, 2012c; Emler, 1994). Therefore, unless the sender’s motive is prosocial (see Beersma & Van Kleef, 2012; Wilson et al., 2000), typical negative gossip such as dating failure (Peters & Kashima, 2014) is likely to undermine perceptions of the gossip sender’s integrity (Michelson et al., 2010).

Consistent with this logic, the ethics and moral psychology literature suggests that morality plays a primary role in how we evaluate individuals (Brambilla & Leach, 2014). In effect, people make quick judgments about others’ morality based on minimal information; they may engage in moral condemnation of others’ behavior even when they perceive it as minimally harmful (see Brambilla & Leach, 2014). Similarly, research has shown that people base their evaluations of others more on moral characteristics like honesty than on non-moral characteristics like competence and that these evaluations are important in forming overall impressions of other people (Pagliaro, et al., 2013).

We suggest that when a gossip sender shares negative information about a target, gossip recipients try to determine whether the sender’s behavior is morally wrong (Ditto & Liu, 2011; Haidt, 2001) and evaluate the sender based on moral characteristics. To the extent that typical negative gossip is perceived as potentially harmful (Emler, 1994; Giardini, 2012; Tebbutt, 1995), we expect gossip recipients to perceive the sender’s morality as low (see Fig. 1).

#### Hypothesis 1

Negative workplace gossip about a peer will result in lower perceived morality of the gossip sender by the gossip recipient.

### Moderating Effect of the Gossip Recipient’s Gender

We next suggest that the aforementioned effects of negative workplace gossip are contingent on the recipient’s gender. This prediction does not benefit from a clear direction in the literature. On the one hand, role congruity theory suggests that men and women act in congruence with gender role stereotypes (Eagly, 1987; Eagly & Steffen, 1984). As individuals share beliefs regarding how men and women should think and behave (Burgess & Borgida, 1999; Eagly & Karau, 2002), conventional gender roles describe women as communal—warm and empathetic—and men as agentic—decisive and forceful (Abele, 2003; Fiske & Stevens, 1993). In line with these behavioral characteristics, we might expect women to rate the gossip sender’s morality less strictly than men. However, empirical findings do not unequivocally support this theoretical prediction: Whereas some studies have found that women express more positive attitudes toward gossip than men (e.g., Leaper & Holliday, 1995), these attitudinal differences have been mostly small in magnitude, with no consistent trend across various studies (see Litman & Pezzo, 2005).

On the other hand, gender socialization theory (e.g., Eagly & Wood, 1991) indicates that women’s communal nature leads them to consider morality as integral to their self-esteem (Ward & King, 2018; Witt & Wood, 2010) and to take a stricter ethical stand (Mason & Mudrack, 1996; Weeks et al., 1999). Consistent with this logic, the literature on gender differences in recognizing (Khazanchi, 1995) and reporting unethical behavior (Stylianou et al., 2013) has shown that women are more concerned with harm and fairness than men (Graham et al., 2011). Similarly, research has shown that women have a stronger preference for deontological judgments than men (Friesdorf et al., 2015). Such judgments are based on affective processing and focus more on morality and consistency of action with moral norms. This difference is enhanced specifically when the deontological judgment requires one to refrain from harmful action (Armstrong et al., 2019). In this sense, women experience stronger affective reactions when perceiving harm caused by any action. Accordingly, research has shown that, in situations with compromised ethical values, women report stronger feelings of moral outrage than men (Kennedy & Kray, 2014) and condemn immoral actions more than men (Ward & King, 2018).

As typical negative gossip may cause harm to the target’s reputation (Emler, 1994; Giardini, 2012; Tebbutt, 1995) thus violating social norms, we expect women to morally condemn the gossip sender more severely than men (See Fig. 1).

#### Hypothesis 2

The relationship between negative workplace gossip about a peer and the perceived morality of the gossip sender will be moderated by the gossip recipient’s gender, such that the negative judgment will be stronger for women than for men.

### Sanctioning Behavior Toward the Gossip Sender

Individuals’ impressions of others’ moral characteristics form the basis of subsequent behavior (Pagliaro et al., 2013). Gossip recipients are thus likely to act in accordance with their negative perceptions of the gossip sender’s morality.

Such action toward the gossip sender can take the form of sanctioning behavior, which refers to penalties and punishments (Mulder et al., 2009; Nelissen & Mulder, 2013; Smith et al., 2007). According to the sanctioning literature, individuals may police and monitor deviant behavior and engage in sanctions to punish those who act in such a deviant way (Horne, 2004), deter them from exhibiting undesirable behaviors, and push them to act in accordance with social norms (Smith et al., 2007). To the extent that negative workplace gossip is perceived as morally wrong and a violation of norms, it is likely to elicit work-related sanctioning behavior. In support of this argument, research suggests that the recipients of negative gossip may feel hostility toward the purveyor of the information (Rosnow, 1988); they may even punish the gossip sender by withholding cooperation or reporting this malevolent behavior to others (Giardini, 2012). In addition, gossiping activity has been negatively related to senders’ supervisor-rated performance (Grosser et al., 2010) and to peer-rated in-role performance (Brady et al., 2017).

Therefore, we propose that attributions of morality translate into sanctioning behavior: Gossip recipients rate senders as low in morality and consequently ‘punish’ their behavior with accessible career and social sanctions, such as low performance ratings, unfavorable feedback for promotions and bonuses, and social exclusion (see Fig. 1). In line with our hypothesis that the negative judgment of the gossip sender’s morality will be stronger for female than for male recipients, we predict a moderated mediation effect as follows:

#### Hypothesis 3

Negative workplace gossip about a peer will result in more career-related and social sanctions toward the gossip sender by the gossip recipient via his or her perception of the gossip sender’s morality. This mediated relationship will be moderated by the gossip recipient’s gender, such that negative responses will be stronger for women than for men.

## Overview of Studies

We tested the above hypotheses in three studies with experimental and field data.^1^ In all cases, respondents were US citizens employed (part- or full-time) at the time of the study, recruited via Qualtrics. For each of the three studies, the instrument was sent via an online link and participation was voluntary. In Study 1, we experimentally tested the impact of negative gossip on moral attributions, as well as the moderating effect of the recipient’s gender. In Study 2, we experimentally examined the effects of negative gossip on perceived morality and, in turn, on career sanctions, and tested the effects of the gossip sender’s gender and the gossip’s work-relatedness (Kurland & Pelled, 2000). Finally, Study 3 generalized our findings by examining negative workplace gossip events reported by a sample of employed individuals using the critical incident technique. This study expanded the outcomes studied to consider the social exclusion of the sender. Taken together, our studies demonstrate a wide array of gossiping behaviors and their consequent impact, shedding light on how gossip senders are perceived and sanctioned by their peers.

## Study 1

### Sample and Procedure

A sample of 179 individuals (average age = 38, SD = 10.86; 46% female) participated in the study.^2^ All participants were assured anonymity and confidentiality and provided full consent. Based on the findings from an exploratory qualitative study designed to gain insight into typical gossip content, we created a scenario of negative gossip about a peer’s personal affairs in a work setting.^3^ We pre-tested the scenario with a sample of business students taking a leadership class, in exchange for extra credit.^4^ Participants were randomly assigned to either the gossip or the no-gossip condition. In the gossip condition, participants read:

Imagine Pat Hill, your coworker, spots you in the hallway as you are walking from another department. Pat comes over to you to say ‘hi’ as usual. After Pat says hello and asks how things are going, Pat leans in closer to you and says something about another employee. Pat says, 'You know the conference we just got back from? Well, our accountant was not working all the time, apparently. I observed a lot of flirting with our marketing director, who already has a partner by the way. And they were seen leaving together very late that evening. Sounds like a secret love story, if you know what I mean!’ After commenting, Pat is called away by someone else across the hall.

In the control condition, participants read the introductory part of the above scenario, and then read:

After Pat says hello and asks how things are going, you two start talking about your project together. Pat says, *'Have you realized that our presentation at the Annual Strategic Meeting is two weeks away?'* You then keep chatting for 5 minutes about the different parts of the presentation and how the accountant can also help you finish things on time.

Next, participants completed a short survey assessing morality, the manipulation check, and demographic characteristics.

### Measures

#### Dependent Variable: Perceived Morality

Using the morality scale by Leach et al. (2007), participants rated the extent to which Pat was “sincere,” “trustworthy,” “honest,” and “respectful” (1 = *does not apply to Pat at all* to 7 = *does apply to Pat extremely well*) (*α* = 0.93).

#### Moderating Variable: Gender

Gender was coded as binary (1 = *female*; 0 = *male*).

#### Control Variables

Because previous research has shown that gossip and moral judgments may depend on age, education, and job level (Brady et al., 2017; Kawamoto et al., 2019; Massar et al., 2012; Neesham & Gu, 2015), we controlled for those variables in our models. *Education level* was measured in years. *Management level* was a dummy variable where 1 signified holding a managerial position.

### Results

#### Manipulation Check

We assessed the efficacy of the gossip scenario on a 7-point Likert scale ranging from 1 (*not at all*) to 7 (*extremely*): “To what extent was the information shared gossip about the accountant?” The analysis showed that participants in the gossip condition reported higher levels of gossip (*M* = 4.899, SD = 1.99) than those in the control condition (*M* = 2.91, SD = 1.77; *t*(177) = 6.95, *p* = 0.001).

#### Hypothesis Testing

Descriptive statistics and correlations for all variables are presented in Table 1. Consistent with previous findings on gender inequality in education and managerial positions in favor of men (e.g., Eagly et al., 2007; Wright et al., 2015), gender was significantly correlated with education (*r* =  − 0.34, *p* < 0.001) and management level (*r* =  − 0.41, *p* < 0.001) in our data.

**Table 1:** Descriptive statistics and correlations for Study 1 variables

		*M*	SD	*α*	1	2	3	4
1	Perceived morality	4.47	1.78	0.93				
2	Gender	0.46	0.50		− 0.27**			
3	Age	37.88	10.86		0.11	− 0.06		
4	Education level	15.78	2.66		0.14	− 0.34**	0.06	
5	Management level	0.69	0.47		0.12	− 0.41**	0.06	0.48**

*N* = 179

*p < .05. **p < .01

ANOVA results showed that participants in the gossip condition reported significantly lower levels of morality (*M* = 3.82, SD = 1.87) than those in the control condition (*M* = 5.25, SD = 1.29, *F*(1,177) = 34.46, *p* = 0.001). ANCOVA results, in which we entered the control variables as covariates, also indicated a significant effect of the gossip condition on perceived morality (*F*(1, 173) = 47.19, *p* < 0.001,* η*^2^ = 0.21). Thus, Hypothesis 1 was supported.

**Fig. 2:**
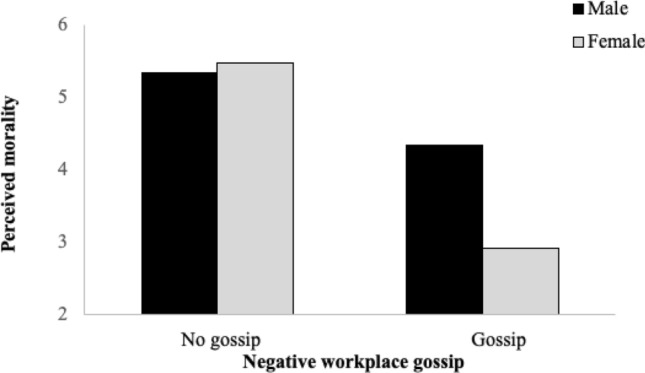
Moderating effect of gossip recipient’s gender on the relationship between negative workplace gossip and perceived morality (Study 1). *Note* Conditional effects of gossip on perceived morality: 95% CI [− 1.63, − 0.36] for men and [− 3.25, − 1.88] for women

We tested Hypothesis 2 in a regression model using the PROCESS macro by Hayes (2017) with Model 1, entering the gossip condition as the independent variable, morality as the dependent variable, gender of participants as the moderator, and the control variables as covariates. The results indicated a significant effect of gossip on the perceived morality of the gossip sender moderated by the recipient's gender (*b* =  − 1.57, SE = 0.45; *F*(1, 172) = 12.17, *p* = 0.001). Simple slopes analysis showed that the effect of gossip on morality was significantly stronger for females (*b* =  − 2.57, SE = 0.35; [− 3.25, − 1.88]) than for males (*b* =  − 0.99, SE = 0.32; [− 1.63, − 0.36]). Visual examination of the plot reproducing these effects (Fig. 2) confirmed that women in the gossip condition rated the gossip sender’s morality more negatively than men. Thus, Hypothesis 2 was supported.

### Brief Discussion

Study 1 supported our prediction that negative gossip about a peer would result in negative perceptions of the gossip sender’s morality. Furthermore, this study provided evidence that the gossip recipient’s gender moderates this relationship, with women rating the sender’s morality more negatively than men.^5^ However, our study design did not enable us to examine the potential role of the gossip sender’s gender. Because previous research has found that people are more interested in gossip about same-sex others than about opposite-sex others (McAndrew et al., 2007), recipients’ reactions may similarly be influenced by their gender similarity with the sender. In addition, our gossip scenario described a peer’s love affair. However, previous research has proposed that gossip about ‘professional’ topics such as a person’s salary might have more relevance than ‘social’ topics such as divorce (Kurland & Pelled, 2000); empirical research on the differential effects of work- vs. non-work-related gossip is nonetheless limited (Kuo et al., 2015).

To address these limitations and test our full mediation model, we conducted our second study.

## Study 2

### Sample and Procedures

A total of 530 paid respondents from Qualtrics participated in the study (51% female, average age 39.88 years [SD = 11.31]). We randomly assigned participants to one of six conditions in a 2 (female vs. male gossip sender) X 3 (work-related vs. non-work-related gossip vs. no gossip) factorial design. For non-work-related gossip and no gossip, we used the scenarios from Study 1, but changed the name of the gossip sender to either “Patrick” or “Patricia” according to the condition. For work-related gossip, participants read the following pilot-tested scenario using “Patrick” or “Patricia” according to the condition:

Imagine Patrick (Patricia) Hill, your coworker, spots you in the hallway as you are walking from another department. (S)he comes over to you to say ‘hi’ as (s)he ordinarily does. After Patrick (Patricia) says hello and asks how things are going, (s)he leans in closer to you and says something about another employee. (S)he says, ‘Did our accountant ever tell you why they left their last job? Well, I’ve heard a few things. Apparently, it was not exactly a resignation. There was a huge scandal about being absent and late all the time. I have also seen this person spending most of the day here on personal calls and emails!’ After commenting, Patrick (Patricia) is called away by someone else across the hall.

Next, participants completed a short survey assessing perceived morality, outcome and control variables, as well as the manipulation check.

### Measures

#### Mediating and Dependent Variables

We measured *perceived morality* using the same scale as in Study 1 (*α* = 0.95).

To measure *career-related sanctions,* we reverse-scored each of the following three measures. *Recommendation for a bonus* was measured with the following item: “In your company, career decisions (bonus, performance, promotions) are partly based on peer evaluations. A few days later, in your role as Patrick (Patricia)’s coworker, you are in a meeting deciding whether Patrick (Patricia) will get a bonus. Please choose one of the following options:” (1 = *no bonus* to 7 = *full bonus*).

*Performance appraisal* was measured with the following item: “A few weeks later, it is time for the performance appraisals, which are also partly based on peer evaluations. In your role as Patrick (Patricia)’s coworker, how would you assess Patrick (Patricia)’s performance?” (1 = *very poor* to 7 = *exceptional*)*.*

*Recommendation for promotion* was measured with the following item: “In your role as Patrick (Patricia)’s coworker, it is time to make a recommendation about Patrick (Patricia)’s career development in the company. Please indicate your recommendation for a promotion” (1 = *Patrick (Patricia) should be fired* to 7 = *Patrick (Patricia) is ready to be promoted any time at a higher level*).

Because these three items were highly correlated and have not been used in aggregated form in previous research, we evaluated the degree to which they represented a unique factor by performing an exploratory factor analysis (EFA; Thompson, 2004; see also Chung et al., 2017). The results showed that the three items loaded on the same factor explained 80% of the variance, with factor loadings ranging from 0.88 to 0.91. We thus calculated an aggregate measure of career sanctions and used it in all analyses (*α* = 0.87).

#### Control Variables

We controlled for the same variables as in Study 1. To conduct a more conservative test of our hypotheses, we also controlled for participants’ *tendency to gossip* because participants with a higher tendency to gossip may rate the gossip sender more favorably. We used the measure by Nevo et al. (1993) with four items on a 7-point Likert scale, ranging from 1 (*never*) to 7 (*always*). An example item is: “I tend to talk with coworkers about the love affairs of people we know” (*α* = *0.8*0).

### Confirmatory Factor Analysis

Before hypothesis testing, we performed a confirmatory factor analysis (CFA) to establish discriminant validity of our latent variables (Thompson, 2004; see also Chung et al., 2017). We tested a model including all variables separately (*χ*^2^ = 426.828, df = 299, RMSEA = 0.07, CFI = 0.94) against various alternative models, collapsing variables on theoretical grounds. This model provided superior results to alternative models,^6^ demonstrating discriminant validity.

### Results

#### Manipulation Check

We used the same manipulation check as in Study 1 and added one item to assess work-related gossip: “To what extent was the information shared gossip about the accountant’s work matters?” and one item to assess non-work-related gossip: “To what extent was the information shared gossip about the accountant’s personal matters?” Analysis showed that participants in the gossip conditions reported higher levels of gossip (non-work-related: *M* = 5.64, SD = 1.62; work-related: *M* = 5.56, SD = 1.62) than those in the no-gossip condition (*M* = 2.78, SD = 1.76; *F*(1,527) = 341.33, *p* = 0.001). Furthermore, participants in the work-related gossip condition reported higher levels of gossip about work affairs (*M* = 5.57, SD = 1.62) than those in the non-work-related gossip condition (*M* = 2.84, SD = 1.79, *t*(357) = 15.12, *p* = 0.001). Finally, participants in the non-work-related gossip condition reported higher levels of non-work-related gossip (*M* = 4.71, SD = 2.00) than those in the work-related gossip condition (*M* = 2.60, SD = 1.76, *t*(357) = 10.62, *p* = 0.001).

#### Hypothesis Testing

Descriptive statistics and correlations for all variables are presented in Table 2. Similar to Study 1, gender correlated significantly with education (*r* =  − 0.24, *p* < 0.001) and management level (*r* =  − 0.37, *p* < 0.001).

**Table 2:** Descriptive statistics and correlations for Study 2 variables

		*M*	SD	*α*	1	2	3	4	5		6	7
1	Perceived morality	3.93	1.86	0.95								
2	Career sanctions	4.57	1.43	0.87	− 0.68**							
3	Gender	0.51	0.50		− 0.26**	0.13**						
4	Age	39.88	11.31		− 0.15**	0.09*	− 0.03					
5	Education level	15.48	2.60		0.31**	− 0.33**	− 0.24**	0.03				
6	Management level	0.62	0.49		0.34**	− 0.26**	− 0.37**	0.04	0.42**			
7	Tendency to gossip	3.19	1.46	0.80	0.49**	− 0.37**	− 0.26**	− 0.16**		0.32**	0.33**	
8	Gossip sender's gender	0.50	0.50		− 0.02	0.01	0.06	0.01		− 0.05	− 0.06	0.03

*N* = 530

*p < .05. **p < .01

We first assessed whether the gossip sender’s gender and the work-relatedness of the gossip had an effect on perceived morality and career-related sanctions toward the sender. The ANOVA results revealed no significant differences between the male and female gossip senders and between the non-work-related and work-related gossip conditions, respectively. Therefore, in subsequent analyses, we collapsed the work-related and non-work-related gossip conditions and entered the sender’s gender as a control variable.^7^

The ANOVA results using the gossip condition as a dichotomous predictor revealed that participants in the gossip condition reported significantly lower levels of morality (*M* = 3.10, SD = 1.54) than those in the control condition (*M* = 5.53, SD = 1.29, *F*(1,528) = 333.10, *p* = 0.001). Thus, Hypothesis 1 was supported.

**Fig. 3:**
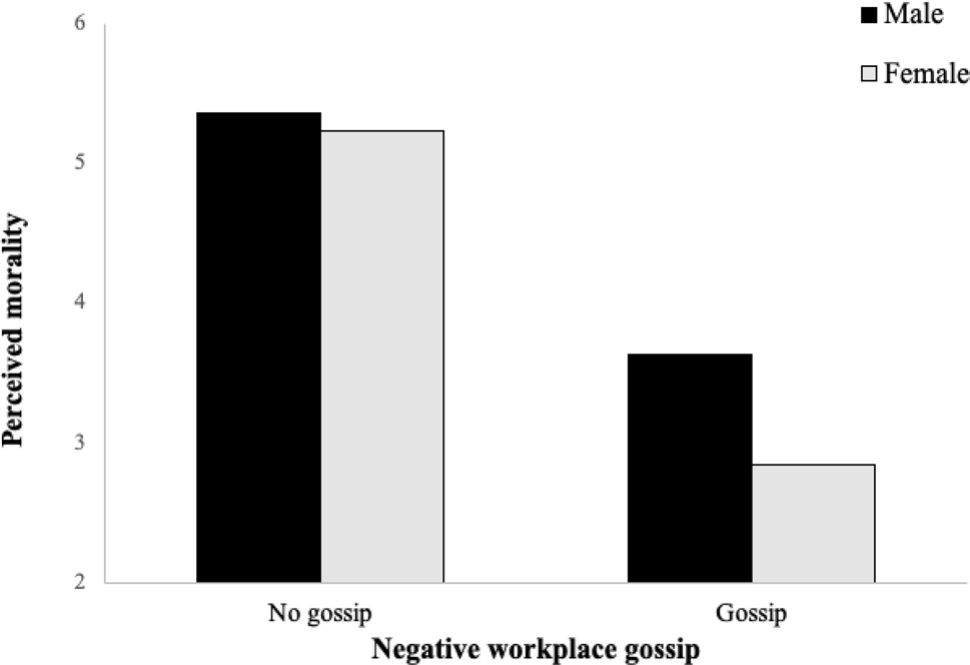
Moderating effect of gossip recipient’s gender on the indirect effect of negative workplace gossip on sanctioning behavior through perceived morality (Study 2). *Note* Conditional effects of gossip on perceived morality: 95% CI [− 2.09, − 1.43] for men and [− 2.75, − 2.09] for women

To test Hypotheses 2 and 3, we ran a moderated mediation analysis using the macro PROCESS by Hayes (2017) with Models 4 and 7. The results with 5,000 bootstrap samples and 95% bias-corrected confidence intervals showed that gossip had an indirect effect on career-related sanctions through perceived morality (*b* = 0.81, SE = 0.09, 95% CI [0.64, 0.99]). The index of moderated mediation for perceived morality was significantly different from zero for career-related sanctions (*b* = 0.25, SE = 0.09; [0.08,0.42]). Additional simple slopes analyses showed that the effect was significant for both genders and significantly higher for women (*b* =  − 2.42, SE = 0.17; [− 2.75, − 2.09]) than for men (*b* =  − 1.76; SE = 0.17; [− 2.09, − 1.43]). Visual examination of the plot reproducing these effects (Fig. 3) revealed that women in the gossip condition rated the gossip sender’s morality more negatively than men. These results supported Hypotheses 2 and 3.

### Brief Discussion

The results of Study 2 supported the idea that negative gossip is viewed as morally wrong through a ‘gendered’ lens, thus replicating the findings from Study 1. They also provided evidence that gossip recipients’ behavioral reactions toward senders can take the form of career-related sanctions. Supplementary analysis using the similarity between the genders of the gossip sender and recipient as a moderator indicated no significant differences. In addition, we found no differences concerning the work-relatedness dimension of gossip.

Although Studies 1 and 2 supported causality, external validity in experimental studies is limited. Furthermore, although previous research has indicated that gossip can facilitate social exclusion (Feinberg et al., 2014), Study 2 did not measure social exclusion as a sanctioning behavior. To address these limitations and replicate and generalize our findings, we conducted an observational study.

## Study 3

### Sample and Procedures

We recruited 229 individuals via Qualtrics. Following Bradfield and Aquino (1999), we used a critical incident technique to gather typical gossip incidents shared by peers at work. Critical incidents enable reflection on a particular workplace episode, ensuring external validity and providing rich data (Chell, 1998). We used a six-month timeframe to increase participants’ recall accuracy. Participants were first asked: “Think back over the last six months as an employee in your current organization to when you experienced gossip behavior by one of your peers. That is, think of an incident when one of your peers informally talked to you about colleague X who was not present. Please write a two- to three-sentence description of the gossip behavior you experienced by your peer.” After describing the gossip incident, participants answered a series of questions about the incident and the gossip sender, as well as about their own behavioral responses.

Before conducting our analyses, two coders assessed the information provided in each reported incident as negative gossip, positive gossip, no gossip, unclear, as well as work-related or non-work-related gossip, following the same procedure as in Study 1 (see Table C in the Supplementary Material). Interrater reliability was excellent (*ICC*(3,2) = 1.00). Out of the 229 reported incidents, 43 referred to situations with no gossip, positive gossip, or unclear information. We thus used the remaining 186 observations (49% female, average age 50.75 years [SD = 12.64]) with negative gossip for all analyses.

### Measure

#### Independent Variable: Gossip Severity

We assessed the perceived severity of the gossip behavior using a single-item index. Such operationalization is consistent with previous research that used critical incidents to measure deviance (see Bradfield & Aquino, 1999). We asked respondents: “How would you rate the peer’s gossip behavior you described” (1 = *not at all serious* to 10 = *extremely serious*).

#### Mediator: Perceived Morality

We measured the perceived morality of the gossip sender by adapting the scale used by Mooijman et al. (2020). Participants indicated how accurately the following three statements (*α* = 0.91) described the gossip sender’s behavior: morally wrong, morally unacceptable, and immoral (1 = *not at all accurate* to 7 = *extremely accurate*).

#### Dependent Variable: Sanctioning Behavior

Following Geddes and Stickney (2011), we measured the recipient’s intentions to sanction the gossip sender through both career-related sanctions and social exclusion.

##### Career-Related Sanctions

Similar to Long and Christian (2015), we asked respondents to indicate the extent to which they were inclined to confidentially recommend a *bonus reduction*, and to provide confidential low *performance ratings* of their peer. We also measured *efforts to impede promotion* by asking participants to rate the extent to which they were inclined to confidentially recommend the demotion of their peer (1 = *not at all* to 7 = *very much; α* = 0.95).

##### Social Exclusion

Participants indicated the extent to which they intended to (a) stop hanging out with their peer as friends; (b) exclude their peer from their Facebook list of friends; (c) stop interacting with their peer about work-related matters; and (d) exclude their peer from their LinkedIn list of contacts on a scale from 1 (*not at all accurate*) to 7 (*extremely accurate*). Items (a) and (b) measured exclusion from the friendship network, whereas items (c) and (d) measured exclusion from the task network. Finally, we measured relational distance with the following item: “I was inclined to distance myself from my peer” (*α* = 0.92).

Similar to Study 2, we conducted an EFA on all dependent variables to evaluate whether career-related sanctions (negative performance evaluation, demotion, bonus reduction) and social exclusion (social exclusion from task and friendship network, relational distance) indicated two overall factors. These results indicated that a two-factor solution explained 91% of the variance (*α* = 0.95).

#### Control Variables

We controlled for the same variables as in Study 2, as well as for participants’ age measured in years. Because this study was conducted during the COVID-19 pandemic, we also controlled for the extent to which participants worked from home (1 = *not at all accurate*, 6 = *between 80 and 100% of my work time*). We controlled for the gossip sender’s gender (1 = *female*, 0 = *male*), their hierarchical level relative to the gossip recipient (0 = *equivalent hierarchical level,* 1 = *higher hierarchical level,* − 1 = *lower hierarchical level*), and the work-relatedness dimension of gossip (1 = *work-related gossip*, 0 = *non-work-related gossip*; *ICC* (3,2) = 1).

#### Social Desirability

We assessed social desirability with the brief social desirability scale (BSDS), a valid, reliable, and non-gender-specific short measure (Haghighat, 2007). The scale comprises four items (1 = *yes*, 0 = *no*). An example item is: “Do you always practice what you preach to people?” Similar to previous research (Kakarika et al., 2022), we examined the correlation between social desirability and the self-reported items. No items showed correlations greater than 0.30 and thus were retained in subsequent analyses.

### Results

#### Confirmatory Factor Analysis

Before hypothesis testing, we performed a CFA to establish discriminant validity among all constructs (Thompson, 2004). We tested a model including all independent, mediator, dependent, and control variables separately (*χ*^2^ = 551.50, df = 299, RMSEA = 0.07, CFI = 0.91) against various alternative models, collapsing variables on theoretical grounds. This model provided superior results to alternative models,^8^ thereby demonstrating discriminant validity.

#### Hypothesis Testing

Descriptive statistics and correlations for all variables are presented in Table 3.

**Table 3:** Descriptive statistics and correlations for Study 3 variables

		*M*	SD	*α*	1	2	3	4	5	6	7	8	9	10	11	12
1	Social exclusion	2.76	1.74	0.92												
2	Career sanctions	1.91	1.57	0.95	0.59**											
3	Workplace gossip	5.98	2.25		0.30**	0.22**										
4	Perceived morality	4.29	1.87	0.91	− 0.49**	− 0.26**	− 0.37**									
5	Gossip work-related	0.66	0.48		− 0.15*	− 0.04	− 0.23**	0.17*								
6	Age	50.75	12.84		− 0.10	− 0.24**	0.09	0.01	− 0.05							
7	Gender	0.49	0.50		− 0.09	− 0.14	− 0.03	0.10	− 0.02	0.12						
8	Education level	15.59	2.47		0.12	0.26**	0.10	− 0.01	0.03	− 0.16*	− 0.27**					
9	Management level	0.47	0.50		0.12	0.26**	0.21**	− 0.22**	− 0.19**	0.01	− 0.30**	0.22**				
10	Telework	3.02	2.09		0.04	0.13	− 0.03	− 0.03	0.07	− 0.05	− 0.21**	0.40**	0.19**			
11	Tendency to gossip	2.85	1.57	0.90	− 0.16*	0.02	− 0.12	0.06	0.07	− 0.10	− 0.05	− 0.02	− 0.05	− 0.01		
12	Gossip sender's gender	0.60	0.49		− 0.01	− 0.08	0.01	0.09	− 0.01	0.02	0.55**	− 0.07	− 0.20**	− 0.01	− 0.13	
13	Gossip sender's hierarchical level	− 0.37	0.75		− 0.02	− 0.02	− 0.04	− 0.01	0.02	0.15*	− 0.02	− 0.17*	0.06	− 0.05	− 0.01	− 0.05

*N* = 229

*p < .05. **p < .01

**Fig. 4:**
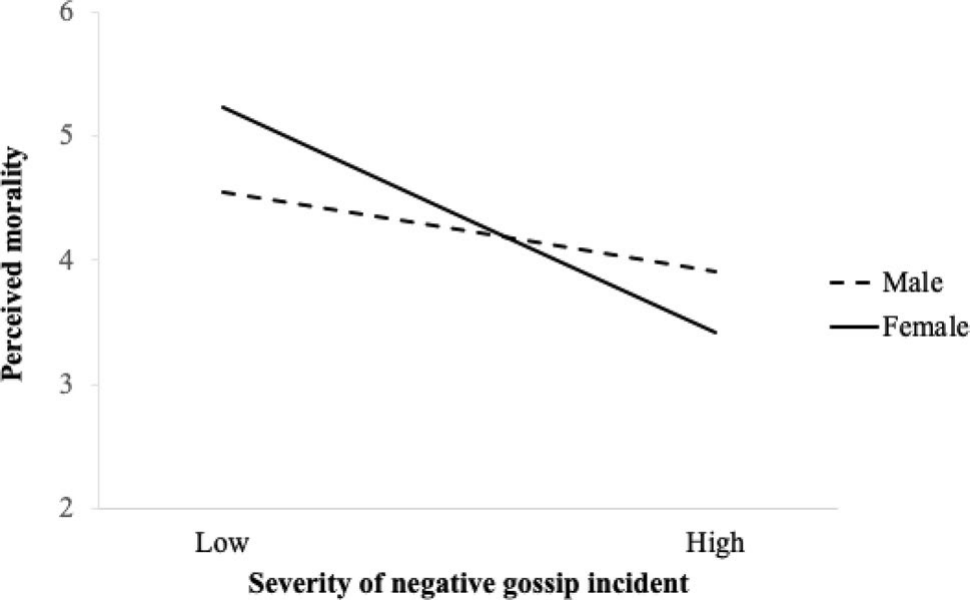
Moderating effect of gossip recipient’s gender on the indirect effect of negative workplace gossip on sanctioning behavior through perceived morality (Study 3). *Note* Conditional effects of gossip severity on perceived morality: 95% CI [− .31, .03] for men and [− .56, − .24] for women

We followed the same procedure as in Study 2 to test our hypotheses. The results of a mediation analysis indicated a significant indirect effect of gossip on career-related sanctions through perceived morality (*b* = 0.04, SE = 0.02, [0.01, 0.09]), as well as on social exclusion (*b* = 0.11, SE = 0.03, [0.05, 0.19]). The moderated mediation results also revealed that these indirect effects were moderated by participants’ gender (career-related sanctions: *b* = 0.04, SE = 0.03, [0.00, 0.11]; social exclusion: *b* = 0.10, SE = 0.06, [0.00, 0.23].) The simple slopes analysis showed that this effect was significant for women (*b* = − 0.40, SE = 0.08, [− 0.56, − 0.24]), but not for men (*b* =  − 0.14, SE = 0.09, [− 0.31, 0.03]) (Fig. 4).^9^

### Brief Discussion

The results of this field study confirmed that recipients of negative gossip perceive senders as low in morality and, in turn, sanction them professionally by being inclined to undermine their promotion, performance appraisal, and bonus. In addition, this study revealed the relational implications of negative gossip for the sender, highlighting social exclusion as an important consequence. Confirming the results of Studies 1 and 2, the effect of negative gossip on attributions of morality was stronger for female than for male recipients.

## General Discussion

We developed and tested a theoretical model to explain how negative gossip about a peer influences the recipient’s judgment of the gossip sender’s morality; this judgment, in turn, drives the recipient’s behavior toward the sender. In three studies with both experimental and observational data, we found robust evidence that the recipients’ negative perceptions of the gossip senders’ morality translate into their willingness to sanction the gossip sender with both career-related penalties and social exclusion. Furthermore, the gossip recipient’s gender shapes the effects of negative gossip on these outcomes. Taken together, our findings support a morality-based and ‘gendered’ approach to negative workplace gossip, underscoring the importance of gossip recipients’ responses and uncovering an important mechanism that has been previously overlooked.

### Contributions to Theory

The first contribution of our paper is to the small but growing body of literature on workplace gossip (e.g., Foster, 2004; Liu et al., 2019) by advancing our understanding of the gossip recipient’s perspective and its implications for the sender. Although scholars have highlighted the need to examine both gossip senders and gossip recipients (Bai et al., 2020), research has been scarce in this regard. For example, research has found that recipients dislike the senders (Ellwardt et al., 2012a, 2012b, 2012c; Farley, 2011), disapprove of them when their motives are self-serving rather than group-serving (see Beersma & Van Kleef, 2012; Wilson et al., 2000), and morally condemn them when the information on the target is not perceived as useful (Peters & Kashima, 2014, 2015). In this narrow body of research, the focus has been on the sender’s prosocial motives and gossip’s utility as perceived by the recipient. In our paper, we advance the understanding of negative workplace gossip by unpacking how the gossip sender is perceived by the recipient, regardless of the motive or perceived usefulness of the conveyed information. In doing so, our studies offer a broader and more realistic representation of gossip behavior and its damage to senders (Lee & Barnes, 2021), beyond extreme negative cases (Brady et al., 2017) and norm violations (e.g., Dores Cruz et al., 2019). By integrating the streams of literature on gossip and moral psychology from an attributional perspective, our findings show that negative workplace gossip about a peer is morally charged.

Importantly, our study identifies gender as a boundary condition on how negative gossip shapes the recipients’ responses to the sender. Previous research has mainly focused on gender differences in the frequency and type of gossip (e.g., Eckhaus & Ben-Hador, 2019; Michelson & Mouly, 2000; Nevo et al., 1993). To our knowledge, no research to date has modeled the gossip recipient’s gender as a moderator. Contrary to previous findings showing that women tend to express more positive attitudes toward gossip than men (Leaper & Holliday, 1995), we found that women are stricter than men when it comes to morally condemning the gossip sender. Our findings that men and women react differently to negative gossip at work have interesting implications for research on gender role expectations (Eagly, 1987) and gender differences in moral reasoning (Friesdorf et al., 2015). In effect, our study clarifies the impact of the recipient’s gender on morality attributions and challenges previous assumptions about women’s positive attitudes toward gossip.

We further contribute to the limited research on the consequences of gossip for the sender by accounting for the gossip recipient’s perspective in sanctioning behavior. Although the link between gossip, networks (Ellwardt et al., 2012a, 2012b, 2012c; Ellwardt et al., 2012a, 2012b, 2012c; Grosser et al., 2010), and reputation (Zinko et al., 2017) has been documented, extant research has not examined the prevalent phenomenon of peer sanctions related to career. This gap in the literature assumes that such consequences stem only from supervisors (see Grosser et al., 2010), as career-related decisions are a typical part of their role responsibilities. Our study moves beyond this assumption and develops a theoretical model of how peers who experience negative gossip may sanction the gossip senders with both career-related penalties and social exclusion. Our work therefore extends sanctioning frameworks by including peer sanctions in the spectrum of the gossip recipients’ behavioral responses, thus underscoring the detrimental consequences for the gossip sender that are dependent on the recipient.

### Practical Implications

Gossip at work has important implications for the employees and for the organization. Our findings suggest that organizations and employees should be vigilant to the incidents of peer gossip and its consequences. First, although it may be challenging for organizations to regulate workplace gossip due to its informal and pervasive nature, they may proactively avoid such negative consequences by raising employee awareness regarding workplace gossip and its moral dimensions. Second, organizations need to be even more vigilant about how recipients’ gender influences their responses to negative workplace gossip and shapes interpersonal dynamics; providing opportunities for men and women to discuss their interpretations of workplace gossip behaviors may reduce strict reactions at work. Finally, employees may recognize the risks associated with peer-to-peer gossip in terms of the recipients’ interpretation and the disproportionately negative career-related penalties and social exclusion. They may benefit from programs targeting interpersonal sensitivity to regulate their responses to negative gossip.

### Limitations and Future Research Directions

Despite these contributions, our studies have limitations that offer several opportunities for future research. One such limitation is that our experimental studies did not enable us to capture the quality of the relationship between the gossip sender and the gossip recipient. In these studies, we described the gossip sender as a coworker without specifying any previous friendship. However, we acknowledge that gossip depends on the recipient–sender interdependence (see Giardini & Wittek, 2019), requiring a degree of discretion between the gossip sender and the gossip recipient (Bergmann, 1993; Spacks, 1982), and that people who are friends at work may be more inclined to gossip with (Grosser et al., 2010; Watson, 2012) and trust each other (Kurland & Pelled, 2000). Therefore, reactions to the experience might differ depending on the nature of the relationship with the gossip sender; people may react less negatively when the gossip sender is a close friend. Their reactions may also vary based on their own moral reasoning. More research is needed to understand how negative gossip travels through social networks and whether our model is moderated by the nature of prior relationships with the gossip sender and the recipient’s moral identity.

Furthermore, although our studies address calls to examine the mechanisms of behavioral responses to negative gossip (Lee & Barnes, 2021), our approach offers only a partial explanation. It may be that other factors play a role in the gossip-sanctioning relationship. Affective events theory (Weiss & Cropanzano, 1996) suggests that emotional reactions to workplace events elicit behavioral responses. Consistent with this theory, responses to the open-ended questions of our pilot study hint that emotional reactions may be important. For example, a participant reported: “…this gossip incident made me feel … uncomfortable … it was making me angry the way they talked about people.” Along these lines, Martinescu et al. (2019) have shown that negative gossip generates negative emotions, which predict retaliation intentions against the sender. In addition, certain gossip episodes may be less morally charged, as recipients may attribute negative gossip to benevolent motives (Lee & Barnes, 2021). For example, the sender may try to condemn a norm violation (Beersma & Van Kleef, 2012; Eriksson et al., 2021), which may be beneficial to the recipient who, in turn, may punish those who do not pass on the group-serving gossip (Wilson et al., 2000). We thus encourage scholars to examine in more detail alternative emotion-based theories and mechanisms, as well as to manipulate the senders’ motives (for a motives typology see Hartung et al., 2019) in order to offer additional explanations for our proposed relationships.

Furthermore, previous research has shown that gossip recipients often reciprocate with gossip of their own (Bergmann, 1993). Therefore, perceptual and behavioral reactions toward the gossip sender may vary over time, as the mutual exchange of gossip between recipients and senders causes reputational information to accumulate (Ellwardt et al., 2012a, 2012b, 2012c; Ellwardt et al., 2012a, 2012b, 2012c). Reactions toward the gossip sender may also vary based on the work or cultural context. Although these ideas were beyond the scope of our research, we encourage scholars to test them in future studies using pre-registered longitudinal designs and modeling contextual variables.

Another limitation of our research was that the scenario used in Study 1 may have, to some extent, caused overlap between the facets of non-work-related and work-related gossip by describing a flirting situation that took place during work. To rule out this possibility, we ran supplementary analyses with an independent sample, for which mention of work time was omitted from the scenario. Although we did not find any differences between the two scenarios, we encourage more research that isolates various types of gossip and examines their behavioral consequences.^10^

Finally, although our study shifts the focus to the perspective of the gossip recipient and the implications for the sender by explicitly focusing on peer-to-peer gossip, we did not explicitly consider the role of the sender’s status in our model. That is, we controlled for the recipients’ managerial position in all studies, as well as the hierarchical level of the sender relative to the recipient in Study 3. As previous research has proposed that status may play a role in the attribution processes of recipients (see Lee & Barnes, 2021) and that gossip can be a strategy for status enhancement (McAndrew & Milenkovic, 2002), examining the role of the sender’s social status in recipients’ judgments and behavioral reactions represents a fruitful line of future research inquiry.

## Conclusion

Despite the pervasiveness of negative workplace gossip, there is still much to learn about the experience for the gossip recipient, the consequences for the sender, and its underlying factors. Our work takes a significant step in this direction. For practitioners, the research findings on the interrelationships among negative gossip, gender, and career can help managers and human resources departments to assess what interventions might be most effective in developing positive workplace environments. We hope future research continues to address this ubiquitous but unexplored complex social interaction in the workplace.

## Supplementary Information

Below is the link to the electronic supplementary material.

Supplementary file1 (DOCX 38 kb)

## Footnotes

1 Data from all three studies are available from the authors upon request.

2 A post hoc sensitivity power analysis using G*Power (Faul et al., 2007) indicated that our sample size for Studies 1 and 2 could reliably detect an effect size of 0.27 and 0.19 with 95% power (*α* = 0.05) and two and six groups, respectively. Such effect sizes fall into the zone of desired effects (Hattie, 2008).

3 Using open-ended questions, 59 graduate students of an organizational behavior course (average age: 22, SD = 1.57; 64% female) described in detail an incident of receiving gossip from a peer at work or at school. Two coders, blind to the purpose of the study, independently read a random sample of 15 open-ended responses and identified the common themes of gossip. Disagreements were discussed between the two coders. Subsequently, each of the coders independently completed the coding for the entire sample. Interrater reliability was excellent (ICC(3,2) = 1.00), as in previous studies (e.g., Bledow et al., 2013). The common themes were mainly related to romance/private issues (53%) vs. work affairs (47%), and in most cases reflected negative situations (79%) during work/study time. These responses provided the basis for the experimental scenarios used in Studies 1 and 2.

4 52 participants evaluated the scenario in terms of realism, indicating the extent to which it reflected typical gossip behavior. Results showed that participants in the gossip condition reported higher levels of typical gossip behavior (*M* = 5.17, SD = 1.39) than those in the control condition (*M* = 2.55, SD = 1.60, *t* = 6.31, *p* = 0.001).

5 These results were not dependent on whether participants thought of Pat as female or male.

6 For example, compared to the model that collapsed the dependent and mediator variables into a single outcome factor, the nine-factor model obtained superior results (Δ*χ*^2^ = 218.65, df = 2, *p* = 0.001).

7 Although collapsing experimental conditions in case of null effects is common practice (e.g., Dean, 2004; Levine & Schweitzer, 2015), we re-ran the analyses with the original conditions and obtained similar results (see Tables A and B in the Supplementary Material).

8 For example, compared to the model that collapsed the dependent variables into a single outcome factor, the 13-factor model obtained superior results (Δ*χ*^2^ = 450.13, df = 3, *p* = 0.001).

9 We tested whether the work-relatedness dimension of gossip impacts our results by re-running the model with work-relatedness as an additional moderator. The results of the conditional moderated mediation (Process Macro Model 11) showed that the three-way interaction effect of gossip severity X recipient’s gender X gossip work-relatedness on morality was not significant. Similarly, controlling for recipient’s gender, the two-way interaction effect of gossip severity X work-relatedness on morality was not significant. These results, available in Tables D and E in the Supplementary Material, indicate that gossip work-relatedness did not impact the results of our main model and justified its use as a control variable.

10 Results of this analysis can be found in the Supplementary Material. We thank an anonymous reviewer for encouraging us to examine this possibility.

## References

[CR1] Abele AE (2003). The dynamics of masculine-agentic and feminine-communal traits: Findings from a prospective study. Journal of Personality and Social Psychology.

[CR2] Armstrong J, Friesdorf R, Conway P (2019). Clarifying gender differences in moral dilemma judgments: The complementary roles of harm aversion and action aversion. Social Psychological and Personality Science.

[CR3] Bai Y, Wang J, Chen T, Li F (2020). Learning from supervisor negative gossip: The reflective learning process and performance outcome of employee receivers. Human Relations.

[CR4] Beersma B, Van Kleef GA (2012). Why people gossip: An empirical analysis of social motives, antecedents, and consequences. Journal of Applied Social Psychology.

[CR5] Ben-Ze'ev A, Goodman RF (1994). Good gossip.

[CR6] Bergmann JR (1993). Discreet indiscretions: The social organization of gossip.

[CR7] Bies RJ, Greenberg J, Cropanzano R (2001). Interactional (in)justice: The sacred and the profane. Advances in organizational justice.

[CR8] Bledow RJ, Rosing K, Frese M (2013). A dynamic perspective on affect and creativity. Academy of Management Journal.

[CR9] Bosson JK, Johnson AB, Niederhoffer K, Swann WB (2006). Interpersonal chemistry through negativity: Bonding by sharing negative attitudes about others. Personal Relationships.

[CR10] Bradfield M, Aquino K (1999). The effects of blame attributions and offender likeableness on forgiveness and revenge in the workplace. Journal of Management.

[CR11] Brady DL, Brown DJ, Liang LH (2017). Moving beyond assumptions of deviance: The reconceptualization and measurement of workplace gossip. Journal of Applied Psychology.

[CR12] Brambilla M, Leach CW (2014). On the importance of being moral: The distinctive role of morality in social judgment. Social Cognition.

[CR13] Burgess D, Borgida E (1999). Who women are, who women should be: Descriptive and prescriptive gender stereotyping in sex discrimination. Psychology, Public Policy, and Law.

[CR14] Chell E, Symon G, Cassell C (1998). Critical incident technique. Qualitative methods and analysis in organizational research: A practical guide.

[CR15] Cheng B, Dong Y, Zhang Z, Shaalan A, Guo G, Peng Y (2020). When targets strike back: How negative workplace gossip triggers political acts by employees. Journal of Business Ethics.

[CR16] Chung GH, Choi JN, Du J (2017). Tired of innovations? Learned helplessness and fatigue in the context of continuous streams of innovation implementation. Journal of Organizational Behavior.

[CR17] Dean DH (2004). Perceptions of the ethicality of consumer insurance claim fraud. Journal of Business Ethics.

[CR18] Ditto PH, Liu B, Mikulincer M, Shaver PR (2011). Deontological dissonance and the consequentialist crutch. The social psychology of morality: Exploring the causes of good and evil.

[CR19] Dores Cruz TD, Beersma B, Dijkstra MT, Bechtoldt MN (2019). The bright and dark side of gossip for cooperation in groups. Frontiers in Psychology.

[CR20] Dores Cruz TD, Nieper AS, Testori M, Martinescu E, Beersma B (2021). An integrative definition and framework to study gossip. Group & Organization Management.

[CR21] Duffy MK, Ganster DC, Pagon M (2002). Social undermining in the workplace. Academy of Management Journal.

[CR22] Dunbar RI (2004). Gossip in evolutionary perspective. Review of General Psychology.

[CR23] Eagly AH (1987). Sex differences in social behavior: A social-role interpretation.

[CR24] Eagly AH, Carli LL, Carli LL (2007). Through the labyrinth: The truth about how women become leaders.

[CR25] Eagly AH, Karau SJ (2002). Role congruity theory of prejudice toward female leaders. Psychological Review.

[CR26] Eagly AH, Steffen VJ (1984). Gender stereotypes stem from the distribution of women and men into social roles. Journal of Personality and Social Psychology.

[CR27] Eagly AH, Wood W (1991). Explaining sex differences in social behavior: A meta-analytic perspective. Personality and Social Psychology Bulletin.

[CR28] Eckhaus E, Ben-Hador B (2019). Gossip and gender differences: A content analysis approach. Journal of Gender Studies.

[CR29] Ellwardt L, Labianca GJ, Wittek R (2012). Who are the objects of positive and negative gossip at work?: A social network perspective on workplace gossip. Social Networks.

[CR30] Ellwardt L, Steglich C, Wittek R (2012). The co-evolution of gossip and friendship in workplace social networks. Social Networks.

[CR31] Ellwardt L, Wittek R, Wielers R (2012). Talking about the boss: Effects of generalized and interpersonal trust on workplace gossip. Group & Organization Management.

[CR32] Emler N, Goodman RF, Ben-Ze’ev A (1994). Gossip, reputation, and social adaptation. Good gossip.

[CR33] Eriksson K, Strimling P, Gelfand M, Wu J, Abernathy J, Akotia CS (2021). Perceptions of the appropriate response to norm violation in 57 societies. Nature Communications.

[CR34] Farley SD (2011). Is gossip power? The inverse relationships between gossip, power, and likability. European Journal of Social Psychology.

[CR35] Faul F, Erdfelder E, Lang A-G, Buchner A (2007). G* Power 3: A flexible statistical power analysis program for the social, behavioral, and biomedical sciences. Behavior Research Methods.

[CR36] Feinberg M, Willer R, Schultz M (2014). Gossip and ostracism promote cooperation in groups. Psychological Science.

[CR37] Fiske ST, Stevens LE (1993). What's so special about sex? Gender stereotyping and discrimination.

[CR38] Foster EK (2004). Research on gossip: Taxonomy, methods, and future directions. Review of General Psychology.

[CR39] Friesdorf R, Conway P, Gawronski B (2015). Gender differences in responses to moral dilemmas: A process dissociation analysis. Personality and Social Psychology Bulletin.

[CR40] Funder DC (2004). The personality puzzle.

[CR41] Geddes D, Stickney LT (2011). The trouble with sanctions: Organizational responses to deviant anger displays at work. Human Relations.

[CR42] Giardini F (2012). Deterrence and transmission as mechanisms ensuring reliability of gossip. Cognitive Processing.

[CR43] Giardini F, Wittek RP (2019). Silence is golden: Six reasons inhibiting the spread of third-party gossip. Frontiers in Psychology.

[CR44] Graham J, Nosek BA, Haidt J, Iyer R, Koleva S, Ditto PH (2011). Mapping the moral domain. Journal of Personality and Social Psychology.

[CR45] Grosser T, Lopez-Kidwell V, Labianca G (2010). A social network analysis of positive and negative gossip in organizational life. Group & Organization Management.

[CR46] Haghighat R (2007). The development of the Brief Social Desirability Scale (BSDS). Europe’s Journal of Psychology.

[CR47] Haidt J (2001). The emotional dog and its rational tail: A social intuitionist approach to moral judgment. Psychological Review.

[CR48] Hartung FM, Krohn C, Pirschtat M (2019). Better than its reputation? Gossip and the reasons why we and individuals with “dark” personalities talk about others. Frontiers in Psychology.

[CR49] Hattie J (2008). Visible learning: A synthesis of over 800 meta-analyses relating to achievement.

[CR50] Hauke N, Abele AE (2020). The impact of negative gossip on target and receiver: A “big two” analysis. Basic and Applied Social Psychology.

[CR51] Hayes AF (2017). Introduction to mediation, moderation, and conditional process analysis: A regression-based approach.

[CR52] Heider F (1958). The psychology of interpersonal relations.

[CR53] Horne C (2004). Collective benefits, exchange interests, and norm enforcement. Social Forces.

[CR54] Kakarika M, Lianidou T, Qu Y, Bligh MC (2022). Organizational behaviour in the COVID-19 context: Effects of supervisor-directed deviance on retaliation against subordinates. British Journal of Management.

[CR55] Kawamoto T, Mieda T, Oshio A (2019). Moral foundations and cognitive ability: Results from a Japanese sample. Personality and Individual Differences.

[CR56] Kelley HH, Michela JL (1980). Attribution theory and research. Annual Review of Psychology.

[CR57] Kennedy JA, Kray LJ (2014). Who is willing to sacrifice ethical values for money and social status? Gender differences in reactions to ethical compromises. Social Psychological and Personality Science.

[CR58] Khazanchi D (1995). Unethical behavior in information systems: The gender factor. Journal of Business Ethics.

[CR59] Kniffin KM, Wilson DS (2010). Evolutionary perspectives on workplace gossip: Why and how gossip can serve groups. Group & Organization Management.

[CR60] Kuo C-C, Chang K, Quinton S, Lu C-Y, Lee I (2015). Gossip in the workplace and the implications for HR management: A study of gossip and its relationship to employee cynicism. The International Journal of Human Resource Management.

[CR61] Kurland NB, Pelled LH (2000). Passing the word: Toward a model of gossip and power in the workplace. Academy of Management Review.

[CR62] Leach CW, Ellemers N, Barreto M (2007). Group virtue: The importance of morality (vs. competence and sociability) in the positive evaluation of in-groups. Journal of Personality and Social Psychology.

[CR63] Leaper C, Holliday H (1995). Gossip in same-gender and cross-gender friends' conversations. Personal Relationships.

[CR64] Lee SH, Barnes CM (2021). An attributional process model of workplace gossip. Journal of Applied Psychology.

[CR65] Levin J, Arluke A, Boston MA (1987). The gossip reporter as anthropologist. gossip.

[CR66] Levine EE, Schweitzer ME (2015). Prosocial lies: When deception breeds trust. Organizational Behavior and Human Decision Processes.

[CR67] Litman JA, Pezzo MV (2005). Individual differences in attitudes towards gossip. Personality and Individual Differences.

[CR68] Liu T, Wu L, Yang Y, Jia Y (2019). Work-to-family spillover effects of workplace negative gossip: A mediated moderation model. Frontiers in Psychology.

[CR69] Long EC, Christian MS (2015). Mindfulness buffers retaliatory responses to injustice: A regulatory approach. Journal of Applied Psychology.

[CR70] Martinescu E, Janssen O, Nijstad BA (2014). Tell me the gossip: The self-evaluative function of receiving gossip about others. Personality and Social Psychology Bulletin.

[CR71] Martinescu E, Janssen O, Nijstad BA (2019). Self-evaluative and other-directed emotional and behavioral responses to gossip about the self. Frontiers in Psychology.

[CR72] Mason ES, Mudrack PE (1996). Gender and ethical orientation: A test of gender and occupational socialization theories. Journal of Business Ethics.

[CR73] Massar K, Buunk AP, Rempt S (2012). Age differences in women’s tendency to gossip are mediated by their mate value. Personality and Individual Differences.

[CR74] McAndrew FT, Bell EK, Garcia CM (2007). Who do we tell and whom do we tell on? Gossip as a strategy for status enhancement. Journal of Applied Social Psychology.

[CR75] McAndrew FT, Milenkovic MA (2002). Of tabloids and family secrets: The evolutionary psychology of gossip. Journal of Applied Social Psychology.

[CR76] Michelson G, Mouly S (2000). Rumour and gossip in organisations: A conceptual study. Management Decision.

[CR77] Michelson G, van Iterson A, Waddington K (2010). Gossip in organizations: Contexts, consequences, and controversies. Group & Organization Management.

[CR78] Mooijman M, Kouchaki M, Beall E, Graham J (2020). Power decreases the moral condemnation of disgust-inducing transgressions. Organizational Behavior and Human Decision Processes.

[CR79] Mulder LB, Verboon P, De Cremer D (2009). Sanctions and moral judgments: The moderating effect of sanction severity and trust in authorities. European Journal of Social Psychology.

[CR80] Neesham C, Gu J (2015). Strengthening moral judgment: A moral identity-based leverage strategy in business ethics education. Journal of Business Ethics.

[CR81] Nelissen RM, Mulder LB (2013). What makes a sanction “stick”? The effects of financial and social sanctions on norm compliance. Social Influence.

[CR82] Nevo O, Nevo B, Zehavi AD, Milton MJ (1993). Gossip and counselling: The tendency to gossip and its relation to vocational interests. Counselling Psychology Quarterly.

[CR83] Pagliaro S, Brambilla M, Sacchi S, D’Angelo M, Ellemers N (2013). Initial impressions determine behaviours: Morality predicts the willingness to help newcomers. Journal of Business Ethics.

[CR84] Peters K, Kashima Y, Forgas JP, Vincze O, László J (2014). Gossiping as moral social action: A functionalist account of gossiper perceptions. Social cognition and communication social cognition and communication.

[CR85] Peters K, Kashima Y (2015). Bad habit or social good? How perceptions of gossiper morality are related to gossip content. European Journal of Social Psychology.

[CR86] Robbins ML, Karan A (2020). Who gossips and how in everyday life?. Social Psychological and Personality Science.

[CR87] Robinson SL, Bennett RJ (1995). A typology of deviant workplace behaviors: A multidimensional scaling study. Academy of Management Journal.

[CR88] Rosnow RL (1988). Rumor as communication: A contextualist approach. Journal of Communication.

[CR89] Smith NC, Simpson SS, Huang C-Y (2007). Why managers fail to do the right thing: An empirical study of unethical and illegal conduct. Business Ethics Quarterly.

[CR90] Sommerfeld RD, Krambeck HJ, Milinski M (2008). Multiple gossip statements and their effect on reputation and trustworthiness. Proceedings Biological Sciences.

[CR91] Spacks PM (1982). In praise of gossip. The Hudson Review.

[CR92] Stylianou AC, Winter S, Niu Y, Giacalone RA, Campbell M (2013). Understanding the behavioral intention to report unethical information technology practices: The role of Machiavellianism, gender, and computer expertise. Journal of Business Ethics.

[CR93] Sun T, Schilpzand P, Liu Y (2022). Workplace gossip: An integrative review of its antecedents, functions, and consequences. Journal of Organizational Behavior.

[CR94] Tebbutt M (1995). Women's talk? A social history of "gossip" in working-class neighbourhoods, 1880–1960.

[CR95] Thompson B (2004). Exploratory and confirmatory factor analysis: Understanding concepts and applications. American Psychological Association.

[CR96] Turner MM, Mazur MA, Wendel N, Winslow R (2003). Relational ruin or social glue? The joint effect of relationship type and gossip valence on liking, trust, and expertise. Communication Monographs.

[CR97] Ward SJ, King LA (2018). Gender differences in emotion explain women’s lower immoral intentions and harsher moral condemnation. Personality and Social Psychology Bulletin.

[CR98] Watson DC (2012). Gender differences in gossip and friendship. Sex Roles.

[CR99] Weeks WA, Moore CW, McKinney JA, Longenecker JG (1999). The effects of gender and career stage on ethical judgment. Journal of Business Ethics.

[CR100] Weiss HM, Cropanzano R, Staw BM, Cummings LL (1996). Affective events theory: A theoretical discussion of the structure, causes and consequences of affective experiences at work. Research in organizational behavior: An annual series of analytical essays and critical reviews.

[CR101] Wert SR, Salovey P (2004). A social comparison account of gossip. Review of General Psychology.

[CR102] Wilson DS, Wilczynski C, Wells A, Weiser L, Heyes C, Huber L (2000). Gossip and other aspects of language as group-level adaptations. The evolution of cognition.

[CR103] Witt MG, Wood W (2010). Self-regulation of gendered behavior in everyday life. Sex Roles.

[CR104] Wright DB, Eaton AA, Skagerberg E (2015). Occupational segregation and psychological gender differences: How empathizing and systemizing help explain the distribution of men and women into (some) occupations. Journal of Research in Personality.

[CR105] Wu L-Z, Birtch TA, Chiang FF, Zhang H (2018). Perceptions of negative workplace gossip: A self-consistency theory framework. Journal of Management.

[CR106] Zinko R, Rubin M (2015). Personal reputation and the organization. Journal of Management & Organization.

[CR107] Zinko R, Tuchtan C, Hunt J, Meurs J, Furner C, Prati LM (2017). Gossip: A channel for the development of personal reputation. International Journal of Organizational Analysis.

