# Supplementary material for: Don’t Shoot the Messenger? A Morality- and Gender-Based Model of Reactions to Negative Workplace Gossip
Source: J Bus Ethics. 2023 Mar 4:1–16. Online ahead of print. doi: 10.1007/s10551-023-05355-7 (PMC9985476; doi:10.1007/s10551-023-05355-7)
Supplement: Supplementary file 1 — Supplementary file1 (DOCX 38 kb) [file 10551_2023_5355_MOESM1_ESM.docx]

**Supplementary Material**

**Study 1**

We tested for differences between the two scenarios with a new sample of 201 participants. After removing four people who failed at least one of two attention checks, our final sample was 197 respondents, all of whom were working part- or full-time, and of which 49% were female, thus mirroring the characteristics of the sample we used in Study 1. Results showed that the two manipulations had similar effects. Participants in the new scenario condition reported similar gossip perceptions (*M* = 5.74, *SD* = 1.26) as those in the original scenario condition (*M* = 5.59, *SD* = 1.20; *t*(195) = -0.91, *p* = *ns*).

**Study 2**

**Table A.** Analysis Including the Moderating Effect of the Gossip Sender’s Gender and the Original Gossip Conditions, Controlling for Recipient’s Gender.

|  |  |  |  |  |
| --- | --- | --- | --- | --- |
|  | Career-related Sanctions (CS) | | | |
| Predictor | *Estimate* | *SE* | *LL* | *UL* |
|  |  |  |  |  |
| *Bootstrapping results for indirect effects* |  |  | *95% CI* | |
| Non-work-related gossip X male sender 🡪 morality 🡪 CS | .84 | .11 | .64 | 1.06 |
| Non-work-related gossip X female sender 🡪 morality 🡪 CS | .83 | .11 | .63 | 1.05 |
| Index of moderated mediation of sender's gender for non-work-related gossip | -.004 | .10 | .21 | .20 |
|  |  |  |  |  |
| Work-related gossip X male sender 🡪 morality 🡪 CS | .79 | .10 | .60 | .99 |
| Work-related gossip X female sender 🡪 morality 🡪 CS | .79 | .10 | .59 | .99 |
| Index of moderated mediation of sender's gender for work-related gossip | -.002 | .10 | -.21 | .19 |

**Table B.** Analysis Including the Moderating Effects of the Gossip Sender’s and Recipient’s Gender and the Original Gossip Conditions.

|  |  |  |  |  |
| --- | --- | --- | --- | --- |
|  | Career-related Sanctions (CS) | | | |
| Predictor | *Estimate* | *SE* | *LL* | *UL* |
|  |  |  |  |  |
| *Bootstrapping results for indirect effects* |  |  | *95% CI* | |
| Non-work-related gossip X male sender X male recipient 🡪 morality 🡪 CS | .70 | .12 | .48 | .96 |
| Non-work-related gossip X male sender X female recipient🡪 morality 🡪 CS | .97 | .13 | .71 | 1.23 |
| Non-work-related gossip X female sender X male recipient🡪 morality 🡪 CS | .61 | .12 | .39 | .86 |
| Non-work-related gossip X female sender X female recipient🡪 morality 🡪 CS | .99 | .14 | .73 | 1.26 |
| Index of moderated mediation of sender's and recipient's gender for non-work-related gossip | .11 | .20 | -.28 | .51 |
|  |  |  |  |  |
| Work-related gossip X male sender X male recipient🡪 morality 🡪 CS | .70 | .11 | .48 | .93 |
| Work-related gossip X male sender X female recipient🡪 morality 🡪 CS | .86 | .13 | 0.62 | 1.12 |
| Work-related gossip X female sender X male recipient🡪 morality 🡪 CS | .66 | .13 | .42 | .93 |
| Work-related gossip X female sender X female recipient🡪 morality 🡪 CS | .87 | .13 | .64 | 1.14 |
| Index of moderated mediation of sender's and recipient's gender for work-related gossip | .04 | .20 | -0.35 | 0.43 |
|  |  |  |  |  |

**Study 3**

**Table C.** Code Book and First 15 Incidents Revised for Disagreement.

|  | **Description of event**  (excerpts examples) | **1. Gossip about non-work-related matters:** 1=yes (e.g., "she was flirting with one colleague"), 0=no | **2. Gossip about work-related matters:** 1=yes (e.g., "he came late to work"), 0=no | **3. Gossip about unknown matters:** 1=yes (e.g., "he just spoke about our colleague' behavior"), 0=no | **4. Gossip negative**: 1=yes (e.g., "she said that he was going behind our backs"), 0=no | **5. Gossip positive:** 1=yes (e.g., "she said she is a good employee"), 0=no | **6. Gossip neutral:** 1=yes (e.g., "she talked about our colleague's personal life"), 0=no |
| --- | --- | --- | --- | --- | --- | --- | --- |
| 1 | *One person told me that he felt a female person in our company was flirting with him. He believed that person suggested having an extramarital affair with him. Both parties are married.* | 1 | 0 | 0 | 1 | 0 | 0 |
| 2 | *One of my colleagues informally came to me to tell me about our senior. I didn’t pay much attention though I knew it was unprofessional.* | 0 | 1 | 1 | 1* | 0 | 0 |
| 3 | *About mental health issues of a just promoted colleague to guide respect of his previous friends who are now subordinates.* | 0 | 1 | 0 | 1* | 0 | 0* |
| 4 | *I had a young worker discuss how the person shamed her and made her feel small. The timing of the shaming made it worse..* | 0 | 1* | 1 | 0 | 0 | 0* |
| 5 | *He told me that my other colleague is lazy and that's why he comes to work late. And that he has a lot of girlfriends on which he spends his money on.* | 1 | 1 | 0 | 1 | 0 | 0 |
| 6 | *When in the office! It was the colleague’s personal affair. He talked about how he's cheating on his faithful wife lately and not taking his work so seriously anymore.* | 1 | 1 | 0 | 1 | 0 | 0 |
| 7 | *The employee was making negative comments about how a woman in the department was dressed. She was excessively critical.* | 1* | 0 | 1 | 1 | 0 | 0 |
| 8 | *It was my co-worker talking about another co-worker and how bad her job performance was. How she sucked up to the boss to get what she wants.* | 0 | 1 | 0 | 1 | 0 | 0 |
| 9 | *A coworker complained about another coworker not doing their job, just getting paid to do nothing. Then this person called in sick.* | 0 | 1 | 0 | 1 | 0 | 0 |
| 10 | *Basically, one member of my team approached me and told me how another member isn’t working at home as they were supposed to be and how they tagged up their computer to make it appear they were in fact working the whole time.* | 0 | 1 | 0 | 1 | 0 | 0 |
| 11 | *My coworker basically told me about something another coworker did that got my supervisor angry. He went around and told lots of people while also saying not to tell were we heard it* | 0 | 1 | 1* | 1 | 0 | 0 |
| 12 | *My colleague stopped at my desk to give me daily reports at the end of the shift. She said, did you hear about Sally? I said what about her? She said she heard that Sally had left her husband and was living with some man she had met a few weeks before that. And she found out that she was pregnant, now doesn't know who the father of the baby is.* | 1* | 0 | 0 | 1 | 0 | 0 |
| 13 | *A coworker discussed mistakes that another coworker makes all the time. Also relayed a story about him not reading his email on a regular basis which caused a little scene.* | 0 | 1 | 0 | 1 | 0 | 0 |
| 14 | *That person was making a comment about an affair that person supposedly was having with a lady. It was very negative and based upon a lot of assumed perceptions. I listened to it & blew it off because I didn't care.* | 1 | 0 | 0 | 1 | 0 | 0 |
| 15 | *Another employee expressed concern about another employee’s performance, or lack thereof. Our job is based on technical performance and the person mentioned operates slower than most counterparts.* | 0 | 1 | 0 | 1* | 0 | 0* |

*Note:* * Indicates that the two coders had an initial disagreement with respect to the dimension evaluated (e.g., valence of gossip). These cases were discussed between the two coders, who arrived to the final coding showed in the table, and clarified the criteria to assess each of the remaining narratives.

**Table D.** Analysis with Gossip Work-Relatedness as Additional Moderator.

|  |  |  |  |  |
| --- | --- | --- | --- | --- |
|  | Sanctioning Behavior | | | |
| Predictor | *Estimate* | *SE* | *LL* | *UL* |
| *Bootstrapping results for indirect effects* |  |  | *95% CI* | |
| Gossip severity X recipient’s gender X gossip work-relatedness 🡪 morality | .038 | .25 | -.88 | .53 |
| Index of moderated mediation for gossip severity X recipient’s gender X gossip work-relatedness 🡪 morality 🡪 career-related sanctions | -.01 | .05 | -.12 | .07 |
|  |  |  |  |  |
| Index of moderated mediation for gossip severity X recipient’s gender X gossip work-relatedness 🡪 morality 🡪 social exclusion | -.02 | .11 | -.24 | .20 |
|  |  |  |  |  |
|  |  |  |  |  |

**Table E.** Analysis with Gossip Work-Relatedness as Additional Moderator, Controlling for Recipient’s Gender.

|  |  |  |  |  |
| --- | --- | --- | --- | --- |
|  | Sanctioning Behavior | | | |
| Predictor | *Estimate* | *SE* | *LL* | *UL* |
| *Bootstrapping results for indirect effects* |  |  | *95% CI* | |
| Gossip severity X gossip work-relatedness 🡪 morality | .18 | .12 | -.06 | .18 |
| Index of moderated mediation for gossip severity X gossip work-relatedness 🡪 morality 🡪 career-related sanctions | -.03 | .03 | -.08 | .02 |
|  |  |  |  |  |
| Index of moderated mediation for gossip severity X gossip work-relatedness 🡪 morality 🡪 social exclusion | -.07 | .06 | -.20 | .04 |
|  |  |  |  |  |
